# Clonotype-Resolved Single-Cell Multi-Omics Unlocks the Profile of Tumor-Infiltrating CD39⁺CD8⁺ T Cells and Enables Adoptive Cell Therapy for Solid Tumor

**DOI:** 10.7150/ijbs.130389

**Published:** 2026-02-18

**Authors:** Zihan Zhao, Xiangyu Wu, Qingyang Jin, Xin Yang, Wenjie Zhu, Ning Jiang, Tianyao Liu, Tianhang Li, Feng Fang, Hongqian Guo, Rong Yang

**Affiliations:** 1 Department of Urology, Nanjing Drum Tower Hospital, Affiliated Hospital of Medical School, Nanjing University, Nanjing, 210008, China.; 2 Department of Pharmacology, School of Basic Medical Sciences, Nanjing Medical University, Nanjing, 211166, China.; 3 Department of Genitourinary Oncology, Tianjin Medical University Cancer Institute and Hospital, National Clinical Research Center for Cancer, Key Laboratory of Cancer Prevention and Therapy, Tianjin, Tianjin's Clinical Research Center for Cancer, Tianjin, 300060, China.

**Keywords:** CD39 (ENTPD1), tumor-infiltrating lymphocyte, TIL therapy, adoptive cell therapy, single-cell sequencing analysis, bladder cancer

## Abstract

Tumor-reactive T cells are central to cancer immunotherapy, and immune checkpoint inhibitors (ICIs) have revolutionized treatment by relieving immune suppression on tumor-reactive T cells, yet response rates remain suboptimal. Adoptive T cell therapy can supplement tumor-reactive T cells, but accurately identifying tumor-reactive CD8⁺ T cells within tumor-infiltrating lymphocytes (TILs) remains challenging. CD39 (ENTPD1) is a rate-limiting enzyme in adenosine metabolism, leading to the view that CD39 is associated with immune suppression because of the inhibitory function of adenosine in tumor immunity. However, its role in tumor-reactive CD8⁺ TIL endures as controversial. In this study, we reassess the tumor-reactive potential of CD39⁺CD8⁺ TILs using clonotype-resolved single-cell multi-omics. Compared to CD39⁻CD8⁺ TILs, CD39⁺CD8⁺ TILs exhibited features of proliferation, activation, and T cell-mediated cytotoxicity, alongside reduced TCR clonal diversity and increased TCR clonal expansion, indicating tumor reactivity. TCR-T cells engineered with TCRs from CD39⁺CD8⁺ TILs mediated robust antigen-specific killing *in vitro*. Importantly, reinfusion of CD39⁺CD8⁺ TILs significantly inhibited tumor growth and demonstrated favorable safety *in vivo*. At the patient level, we further demonstrated that CD39⁺CD8⁺ TILs are enriched for effector programs and pathways linked to T-cell activation and cytotoxicity, and exhibit reduced TCR clonal diversity with pronounced clonal expansion. The intratumoral abundance of CD39⁺CD8⁺ TILs also correlated with earlier tumor stage and improved overall survival, and a CD39⁺CD8⁺ TIL-derived gene signature predicted ICI response and prognosis, supporting CD39 as a practical biomarker to enrich tumor-reactive CD8⁺ TILs and to improve adoptive cell transfer strategies in future clinical practice.

## Introduction

Immune checkpoint inhibitor (ICI) therapies, particularly those targeting the PD-1/PD-L1 axis, have significantly advanced cancer treatment by restoring the immune system's ability to recognize and eliminate tumor cells^1^-^3^. These therapies accomplish this by relieving immune suppression of endogenous tumor-reactive T cells, thereby enabling them to mediate tumor-specific cytotoxicity through the T cell receptor-peptide-major histocompatibility complex (TCR-pMHC) interaction^4^-^6^. Despite their success in subsets of solid tumors, the overall response rate to ICIs remains suboptimal, with many patients failing to derive clinical benefit^5^, ^7^. Although this limited efficacy is multifactorial, a contributing factor is likely the insufficient quantity and functionality of tumor-reactive T cells, which limits the therapeutic efficacy of endogenous T cells and restricts the full potential of immune therapies^8^.

To overcome this limitation, adoptive cell therapy (ACT) has emerged as a promising treatment strategy that amplifies anti-tumor immunity by *ex vivo* expanding and reinfusing tumor-reactive T cells^9^. Among these approaches, tumor-infiltrating lymphocyte (TIL) therapy has demonstrated clinical efficacy across multiple solid tumors^10^. However, the clinical success of ACT is constrained by the substantial heterogeneity within TIL populations^11^, ^12^. Even within TILs containing tumor-reactive T cells, a significant fraction comprises bystander T cells that recognize viral or other non-tumor antigens, thereby diluting overall therapeutic potency^13^, ^14^. Therefore, accurately identifying and isolating tumor-reactive T cells within the tumor immune microenvironment (TIME) remains a major unmet need in T cell immunotherapy^15^.

The TIME is a complex ecosystem comprising tumor cells, T cells, B cells, fibroblasts, myeloid cells and other stromal components^16^, ^17^. Immune cell function is regulated not only by phenotype and receptor expression but also by metabolic products^18^. Among these, adenosine metabolism serves as a key regulator of immune responses within the TIME^19^. During tumor progression and tissue stress, extracellular ATP (eATP) is released in large quantities and swiftly converted into adenosine by CD39 and CD73^20^. Adenosine, through its interaction with A2A/A2B receptors, exerts an immunosuppressive effect. Therefore, traditional views associate CD39 with immune suppression due to its role in adenosine production^21^. However, its function in tumor-reactive CD8⁺ T cells remains controversial. Recent studies suggest that CD39 may also be pivotal in enhancing the tumor-reactive potential of CD8⁺ T cells, particularly within tumor-infiltrating contexts^22^-^28^.

**Scheme 1:**
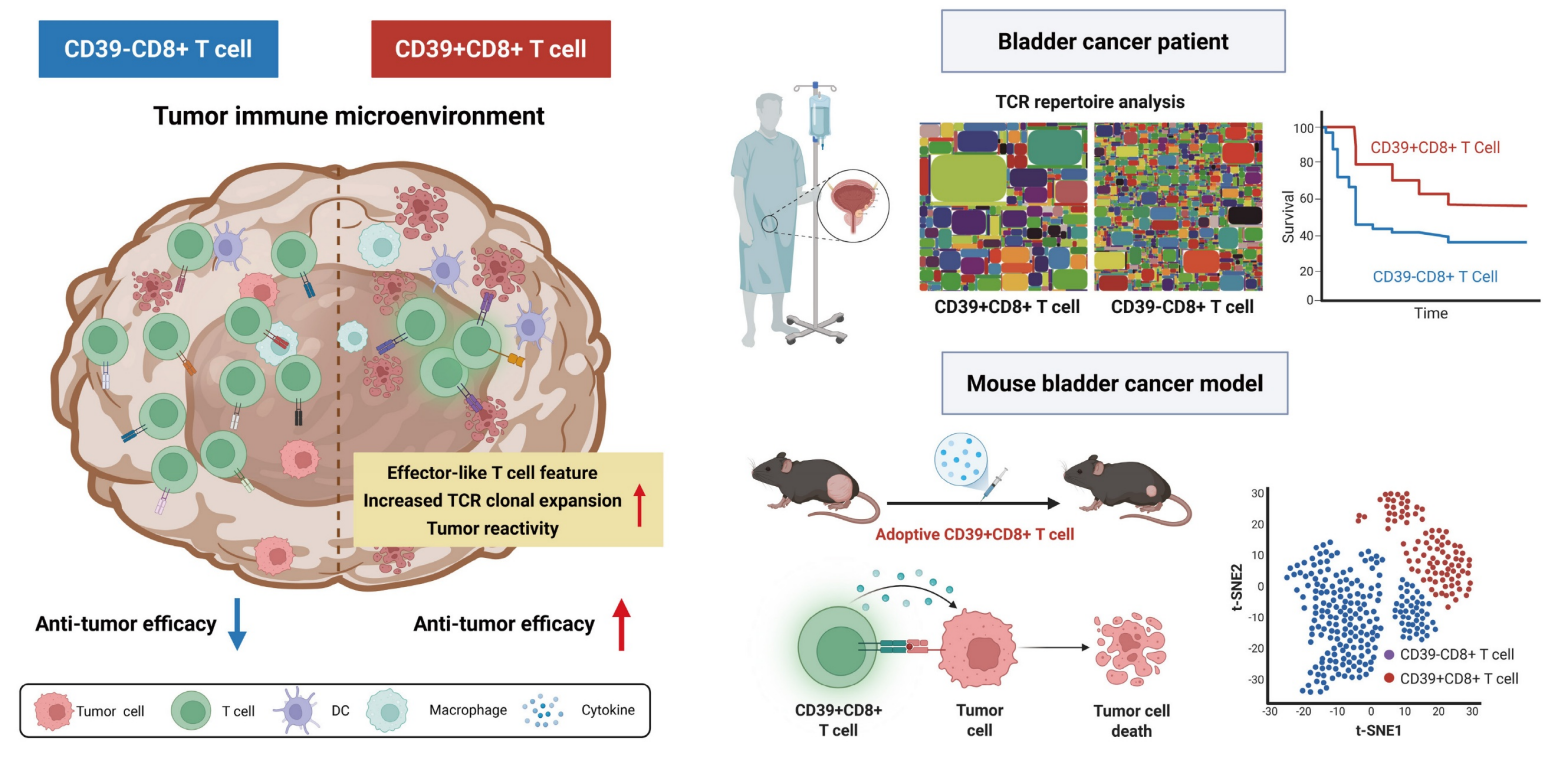
** The roles of CD39⁺CD8⁺ TILs in the tumor immune microenvironment.** The left panel depicts CD39⁺CD8⁺ T cells with features consistent with antitumor activity, including increased TCR clonal expansion and an effector-like feature. The right panel shows that, in bladder cancer patient cohorts, CD39⁺CD8⁺ TILs are associated with clinical outcomes. In parallel, adoptive transfer experiments in a murine bladder cancer model support a functional contribution of CD39⁺CD8⁺ T cells to tumor control. Overall, the scheme highlights CD39 as a practical biomarker to enrich tumor-reactive CD8⁺ TILs and to inform the development of cancer immunotherapy.

Understanding the precise role of CD39 in tumor-reactive CD8⁺ TILs is critical for improving the efficacy of ACT. In this study, we employ clonotype-resolved single-cell multi-omics (paired scRNA-seq and scTCR-seq) to reassess the tumor-reactive potential of CD39⁺CD8⁺ TILs (Scheme 1). We systematically analyze the transcriptional and immune repertoire characteristics of CD39⁺CD8⁺ TILs and quantify their abundance across solid tumor patient cohorts. We found that CD39⁺CD8⁺ TILs exhibit features of proliferation, activation, tumor reactivity, and T cell-mediated cytotoxicity, accompanied by reduced TCR clonal diversity and increased TCR clonal expansion. Adoptive transfer of CD39⁺CD8⁺ TILs significantly suppressed tumor growth and demonstrated a favorable safety profile *in vivo*. Moreover, TCR-T cells expressing TCR from CD39⁺CD8⁺ TILs shown significant activation and killing ability against tumor cells. In bladder cancer (BLCA) patients, CD39⁺CD8⁺ T cell infiltration decreased with increasing tumor stage. Furthermore, CD39⁺CD8⁺ TILs were significantly associated with better overall survival (OS) in three BLCA cohorts, comprising 701 patients. Collectively, our findings define CD39⁺CD8⁺ TILs as a tumor-reactive, clonally expanded T cell subset with translational relevance in bladder cancer, supporting CD39 as a practical marker to enrich anti-tumor T cells for ACT in solid tumors.

## Materials and Methods

### Mice

All experimental animal protocols followed the regulations of the People's Republic of China on the Administration of Laboratory Animals, and all animal procedures in this study were approved by the Animal Experimental Committee of Nanjing Drum Tower Hospital. Six to eight-week-old female C57BL/6J mice were purchased from GemPharmatech (Nanjing, China) and maintained on a 12:12-hour light-dark cycle in a pathogen-free environment, with 5 mice per cage, with ad libitum access to water and food.

### Cell line and culture

The murine bladder cancer cell line MB49 was kindly gifted by Professor Haibo Shen (Shanghai Jiao Tong University, China). MB49 cells were cultured in RPMI 1640 (Gibco) supplemented with 10% fetal bovine serum (FBS, Gibco) and 1× penicillin-streptomycin (Gibco) at 37°C under 5% CO_2_ in a humidified incubator and were routinely tested negative for mycoplasma.

### TILs isolation and culture

1×10^6^ MB49 cells were injected subcutaneously into the right flank of mice to establish a MB49 bladder tumor model. When the average tumor volume reached approximately 500 mm³, the mice were humanely euthanized with CO_2_ followed by cervical dislocation to remove the tumor and purify the TILs. CD8^+^ T cells, CD39^-^CD8^+^ T cells, and CD39^+^CD8^+^ T cells were sorted by a FACS Aria III cell sorter (BD Biosciences). Sorted TILs were cultured in T-cell medium [RPMI 1640 (Gibco) supplemented with 10% FBS (Gibco), 1× penicillin-streptomycin (Gibco), 1× HEPES (Gibco), 1× NEAA (Gibco), 1× sodium pyruvate (Gibco), and 0.1×β-ME (Gibco)], followed by supplementation with 3000 IU/mL interleukin-2 (IL-2) for rapid T cell expansion.

### Systemic adoptive transfer model for TIL therapy

To establish a mouse bladder cancer model, 1×10^6^ MB49 cells were injected subcutaneously into the right flank of mice. When the average tumor volume reached approximately 80 mm³ on day 7, the mice received intraperitoneal injections of cyclophosphamide (CTX, 2mg) for lymphodepletion. Mice were randomly and equally allocated to four experimental groups, including DPBS group, CD8^+^ T cells group, CD39^-^CD8^+^ T cells group, and CD39^+^CD8^+^ T cells group. CD8^+^ T cells, CD39^-^CD8^+^ T cells, and CD39^+^CD8^+^ T cells were respectively administered intravenously in 250 µL DPBS at day 8 and day 15. IL-2 (2.5×10^5^ IU) was given intraperitoneally every 12 hours for 3 days following T cell injections. Tumor volume was measured every 3 days, and the experiment was terminated when the tumor volume reached approximately1000 mm^3^. The mice were humanely euthanized with CO_2_ followed by cervical dislocation.

### Animal model studies

Mouse tumors were fixed in 4% paraformaldehyde for 30 minutes at room temperature or overnight at 4 ℃. Paraffin embedding was performed according to standard protocols. Serial 3 μm sections were cut from paraffin-embedded tissue blocks of mouse tumors. Deparaffinization was performed according to standard protocols. Hematoxylin and eosin (H&E) staining was performed following a standard histologic protocol. For immunohistochemistry (IHC), sections were boiled in 10 mmol/L sodium citrate buffer (pH 6.0) for 5 minutes, cooled, and blocked with 5% bovine serum albumin for 45 minutes. Sections were incubated with CD3ε (D4V8L) rabbit mAb (1:300), anti-CD8 alpha antibody (1:4000), and granzyme B/H antibody (1:6400) overnight at 4°C. Horseradish peroxidase-conjugated anti-rabbit antibodies were used as secondary antibodies. Bound antibody was detected with a DAB detection kit (Abcam, cat#ab64238). To verify the safety of adoptive cell transfer treatment with TILs, we examined H&E-stained slides of liver, lung, and kidney from all groups. In addition, mouse blood was collected to separate serum for liver and renal function marker testing.

### Generation of TCR-Jurkat

The full TCR sequence was selected from the most expanded TCR clone in CD39^+^CD8^+^ TILs by single-cell TCR sequencing (scTCR-seq), and then synthesized and subcloned. Lentivirus was produced in 293T cells and lentiviral supernatant was harvested at 48 hours after transfection. 5 × 10^5^ knockout TCR Jurkat (ΔTCR-Jurkat) were cultured in 24-well plates, TCR lentiviral particles were then added, and the plates were centrifuged at 1000 g for 90 minutes at 30 °C. After centrifugation, the supernatants were removed and replaced with fresh medium. PE anti-mouse TCR β chain antibody (Biolegend, cat#109207) was used to evaluate transduction efficiency after infection.

### Generation of TCR-T cells

Naive CD8^+^ T cells were isolated from C57BL/6J mice using the EasySep mouse CD8^+^ T cell isolation kit (STEMCELL, cat#19853), and activated by plate-bound anti-CD3 (8 μg/mL) and anti-CD28 (2 μg/mL) antibodies for 2 days. 3× 10^5^ activated CD8^+^ T cells were spin-infected for 1.5 hours at 1000 g with 400 µL of viral supernatant in the presence of 8 µg/mL polybrene and then removed from the viral supernatant and resuspended in the indicated T cell culture medium supplemented with 200 IU IL-2, 10 ng/mL IL-7, and 10 ng/mL IL-15. Transduction efficiency was assessed by eGFP expression after infection.

### Activation assay

MB49 tumor cells were seeded into 96-well plates and treated across four gradient conditions. TCR-Jurkat cells were co-cultured with MB49 tumor cells at effector-to-target (E:T) ratios of 1:0, 1:1, 1:2, and 1:5 for 24 hours, with ΔTCR-Jurkat cells serving as the negative control. After 24 hours of co-culture, cells were stained with APC-conjugated anti-human CD69 antibody (Biolegend, cat#310910) and analyzed by flow cytometry. The mouse colorectal cancer CT26 cells, human bladder cancer UMUC3 cells and mouse renal cells were used to evaluate the tumor reactivity of TCR-Jurkat cells. Each condition was performed in three independent biological replicates.

### Cytotoxicity assay

MB49-luc-eGFP tumor cells were seeded in 96-well plates. TCR-T cells were co-cultured with MB49-luc-eGFP tumor cells at effector-to-target (E:T) ratios of 0:1, 1:1, 4:1, and 8:1 for 24 hours, with CD8^+^ T cells serving as a negative control. Following incubation, cell viability was assessed using the Bio-Lite Luciferase Assay Buffer (Vazyme, cat#DD1201). All conditions were tested in three independent biological replicates.

### Single-cell RNA/TCR library preparation and sequencing

After dissociating MB49 subcutaneous tumors in mice into single-cell suspensions, CD39^+^ CD8^+^ T cells and CD39^-^ CD8^+^ T cells were isolated using FACS Aria III cell sorter (BD Biosciences), followed by single-cell RNA sequencing (scRNA-seq) and scTCR-seq.

scRNA-seq library was prepared following the Singleron GEXSCOPE protocol. Single-cell suspensions were captured using the microfluidic chip, and the GEXSCOPE Single-cell RNA Library Kit was used for further processing. This involved generating single-cell gel bead-in-emulsions using a reverse transcription mix and single-cell 3' beads. Subsequent steps included complementary DNA (cDNA) fragmentation, adapter ligation, purification, PCR amplification, fragment selection, and quality control. Libraries were sequenced on the Illumina HiSeq X platform (Illumina, USA).

scTCR-seq library was constructed using the GEXSCOPE Single Cell Immuno-TCR Kit (Singleron, China) according to the manufacturer's instructions. Briefly, the poly(A) tail of mRNAs and the TCR region of T cells were captured by magnetic beads. The mRNA sequencing procedure followed the same steps as mentioned above. mRNAs captured by the magnetic beads were reverse transcribed into cDNA and amplified. Subsequently, the remaining cDNA was enriched for TCR α/β sequences, and PCR amplification was carried out to construct another TCR library, compatible with the Illumina HiSeq X platform.

### Patients

This study included 125 BLCA patients who underwent surgical treatment at Nanjing Drum Tower Hospital (Nanjing, China) from August 2015 to April 2022. The pathological data of 82 patients used for IHC staining were documented in Table S1, and those of another 43 patients used for flow cytometry analysis in Table S2, including age at diagnosis, gender, pathological tumor stage, and tumor grade. Overall survival was defined as the time from the date of surgery to the date of death or to the last follow-up. The study related to human clinical samples was approved by the Ethics Committee of Nanjing Drum Tower Hospital in accordance with the Declaration of Helsinki (2021-394-01, 2024-884-02).

### Immunohistochemistry

For double IHC staining, anti-human CD8 monoclonal antibody (Proteintech, cat#66868-1-Ig) and anti-human CD39 polyclonal antibody (Proteintech, cat#14211-1-AP) were used for IHC staining. Positive cells were quantified as the mean number of stained cells in representative high-power fields (HPF, ×200) across three sections, and the cut-off value was defined as the mean cell number per HPF.

### Flow cytometry

Fresh tumor samples from 43 patients were collected at the time of surgery and preserved in specimen preservation medium for flow cytometry analysis (Table S2). Tumor tissues were then prepared as single-cell suspensions, and Fc receptors were blocked with Human TruStain FcX (Biolegend, cat#422302) before staining with fluorescent antibodies. The Zombie Aqua Fixable Viability Kit was used to distinguish live and dead cells (Biolegend, cat#423102). All fluorescence-labeled antibodies are listed in Table S3 and the gating strategy was shown in Figure S5.

### Bioinformatics analysis

Raw fastq files were processed with CeleScope software (Singleron) to generate gene expression matrices and contig annotations. For functional analysis of mouse CD39⁺CD8⁺ and CD39⁻CD8⁺ T cells, scRNA-seq data were processed in the Seurat R package. High-quality cells (n = 12,400) were filtered with the following criteria: 200-4,000 detected genes and mitochondrial gene expression < 5%, and retained for downstream analysis. After normalization, the top 2,000 highly variable genes (HVGs) were identified using FindVariableFeatures, with dimensionality reduction performed by principal component analysis (PCA) via RunPCA. Cell neighbor relationships and clustering were determined with FindNeighbors and FindClusters, respectively, and cellular distribution was visualized by t-distributed stochastic neighbor embedding (t-SNE) using RunTSNE. All cells were annotated into 11 major cell types according to previously reported annotations^29^. Differentially expressed genes (DEGs) across cell subpopulations were called by Seurat's FindMarkers function. Gene Ontology (GO) functional enrichment analysis was conducted with the clusterProfiler R package^30^, pathway enrichment analysis with the Gene Set Enrichment Analysis (GSEA) package, and gene signature quantification with the UCell R package^31^. Genes included in the gene signature scores are presented in Table S4^32^-^34^. For scTCR-seq analysis, paired transcriptomic and TCR sequencing data were integrated into a single Seurat object, with downstream clonotype analysis performed using the scRepertoire R package^35^.

**Figure 1:**
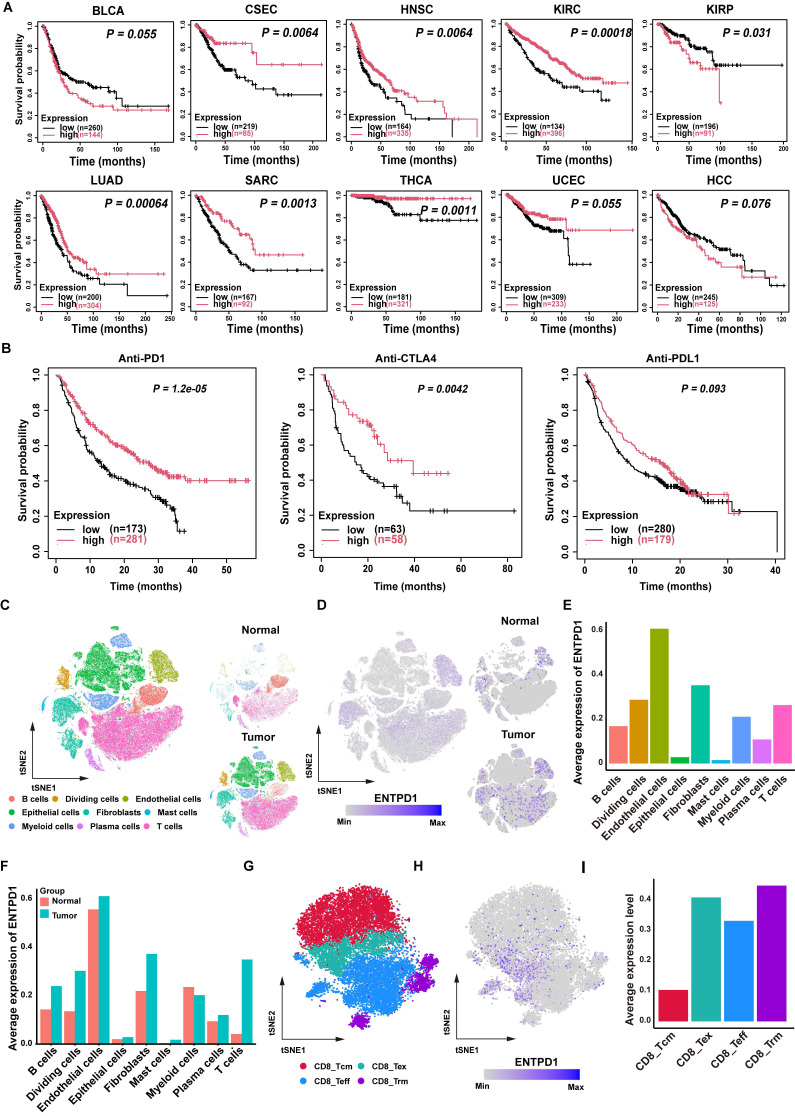
**Clinical prognosis association and single-cell distribution of CD39 (ENTPD1).** (A) Kaplan-Meier analysis of overall survival stratified by ENTPD1 expression across multiple solid tumor types in TCGA. (B) Kaplan-Meier analysis stratified by ENTPD1 expression in patients receiving immune checkpoint blockade. (C) t-SNE of integrated scRNA-seq data from 20 BLCA patients, annotated into 9 major cell types. (D) FeaturePlot of ENTPD1 expression across all cells and split by tissue origin (bladder normal vs tumor). (E) Average ENTPD1 expression across major cell types. (F) Comparison of average ENTPD1 expression between tumor and normal tissues within each major cell type. (G) t-SNE visualization of the CD8^+^ T cell populations from bladder tumor. (H) FeaturePlot of ENTPD1 expression across CD8⁺ T cell subsets in tumor tissue. (I) Average ENTPD1 expression across CD8⁺ T cell subsets.

For the integrated analysis of human BLCA scRNA-seq profiles (Figure 1), five scRNA-seq datasets (GSE222315, GSE146137, GSE130001, GSE149652, GSE176249; 20 patients total) were retrieved from the Gene Expression Omnibus (GEO, https://www.ncbi.nlm.nih.gov/geo/). Raw data were integrated and preprocessed in Seurat with strict quality control filters: cells with < 200 or > 4,500 detected genes, or mitochondrial gene content >10%, were excluded to remove low-quality cells, yielding 126,453 high-quality cells for subsequent analysis. Following normalization, the top 2,000 HVGs were selected via FindVariableFeatures, and PCA was performed for dimensionality reduction using RunPCA. Technical batch effects across datasets were corrected with the Harmony algorithm to eliminate non-biological variation, and t-SNE visualization was generated based on harmonized principal components. All cells were annotated into 9 major cell types by canonical marker gene expression, with annotation results manually validated using DEGs identified by FindMarkers for each cell type.

For the analysis of human CD8⁺ TILs (Figure 6), CD8⁺ TILs from the above five public scRNA-seq datasets were integrated with three in-house BLCA scRNA-seq samples. Batch effects between public and in-house datasets were mitigated via Canonical Correlation Analysis (CCA), with integration anchors identified by FindIntegrationAnchors and a merged expression matrix generated by IntegrateData. Dimensionality reduction and cell clustering were performed by PCA and t-SNE (RunTSNE). Human CD8⁺ TILs were re-annotated for CD39⁺CD8⁺ subsets according to previously reported annotation criteria^29^. DEGs between CD39⁺ and CD39⁻CD8⁺ TIL clusters were called by Seurat's FindMarkers. GO functional enrichment analysis was conducted with ClusterProfiler, pathway enrichment analysis with the GSEA package, and gene signature scoring with UCell. For bulk TCR-seq analysis, TCR clonotype distributions of CD39⁺CD8⁺ and CD39⁻CD8⁺ TILs were visualized as treemaps using the Python squarify library.

For the analysis of CD39⁺CD8⁺ T cell infiltration in clinical cohorts, a CD39⁺CD8⁺ TIL signature was constructed using the mean expression of *CD8A*, *ENTPD1*, *IFNG*, *GZMB*, *CCL4*, *PRF1*, and *CCL3* in the TCGA-BLCA and IMvigor210 cohorts^36^, ^37^. Subsequently, survival analysis was performed based on this signature score.

### Statistical analysis

All values are presented as the mean ± standard error of the mean (SEM), and statistical analyses were performed using GraphPad Prism 8.0. One-way ANOVA was used to assess intergroup statistical significance. Kaplan-Meier analysis was conducted to estimate overall survival probabilities, with differences between groups compared using the log-rank test. Correlations were analyzed via Spearman's rank correlation test, and the associations between biomarkers were determined using Pearson's correlation test. All statistical tests were two-sided, and a P value < 0.05 was considered statistically significant. In all figures, ns, *P* > 0.05; * *P* < 0.05; ** *P* < 0.01; *** *P* < 0.001; **** *P* < 0.0001.

## Results

### CD39 is associated with cancer patients' prognosis and expressed on multiple cell types

To explore the role of CD39 (ENTPD1) in solid tumors, we first analyzed the correlation between CD39 expression and OS across multiple solid tumor types using TCGA data^38^. Interestingly, high CD39 expression was generally associated with better survival outcomes, whereas high CD73 (NT5E, another rate-limiting enzyme catalyzing adenosine production) expression correlated with poorer survival in most cancers, further highlighting the differential roles of the adenosine metabolism pathway in tumor immunity (Figure 1A, Figure S1). In the context of immunotherapy, we found that high CD39 expression was positively correlated with the efficacy of T cell-targeted immune checkpoint inhibitors, such as PD-1 and CTLA-4 inhibitors, but did not correlate with the efficacy of PD-L1 blockade that mainly targets tumor cells (Figure 1B). These data suggest that CD39 may reflect broader T cell immune states beyond its established function as a rate-limiting enzyme in adenosine metabolism, and may be informative for ICIs response.

Previous studies have reported that CD39 is expressed on both cancer cells and immune cells, suggesting a complex expression pattern within the TIME^39^. Therefore, interpreting its association with tumor immunity based solely on bulk TIME expression may be misleading. Instead, the role of CD39 should be re-evaluated at the single-cell level, particularly in T cells. To investigate this further, we compiled single-cell transcriptomic sequencing datasets from five cohorts, including samples from 20 BLCA patients. After quality control and batch effect correction, we obtained a total of 126,453 high-quality single cells, which were categorized into 9 cell clusters: B cells, dividing cells, endothelial cells, epithelial cells, fibroblasts, mast cells, myeloid cells, plasma cells, and T cells (Figure 1C, Figure S2). Based on these datasets, we analyzed CD39 expression across different tissues and cell types. Indeed, CD39 is expressed on various cell types, such as endothelial cells, fibroblasts, and T cells, highlighting the necessity of analyzing CD39 expression at the single-cell level (Figure 1D, 1E).

Compared to normal tissues, CD39 expression is more abundant in tumor tissues, especially in T cells, where the expression of CD39 in TILs is higher than in T cells of normal tissues (Figure 1F). Further analysis revealed that CD39 expression was higher in effector TILs and exhausted TILs (Figure 1G-1I). These findings indicate that CD39 is enriched within tumor-infiltrating T cell states linked to tumor reactivity and support prioritizing CD39⁺CD8⁺ TILs for downstream functional validation and therapeutic exploitation in immunotherapy.

### CD39^+^CD8^+^ TILs exhibit an effector-like functional phenotype and are associated with better prognosis in BLCA patients

**Figure 2:**
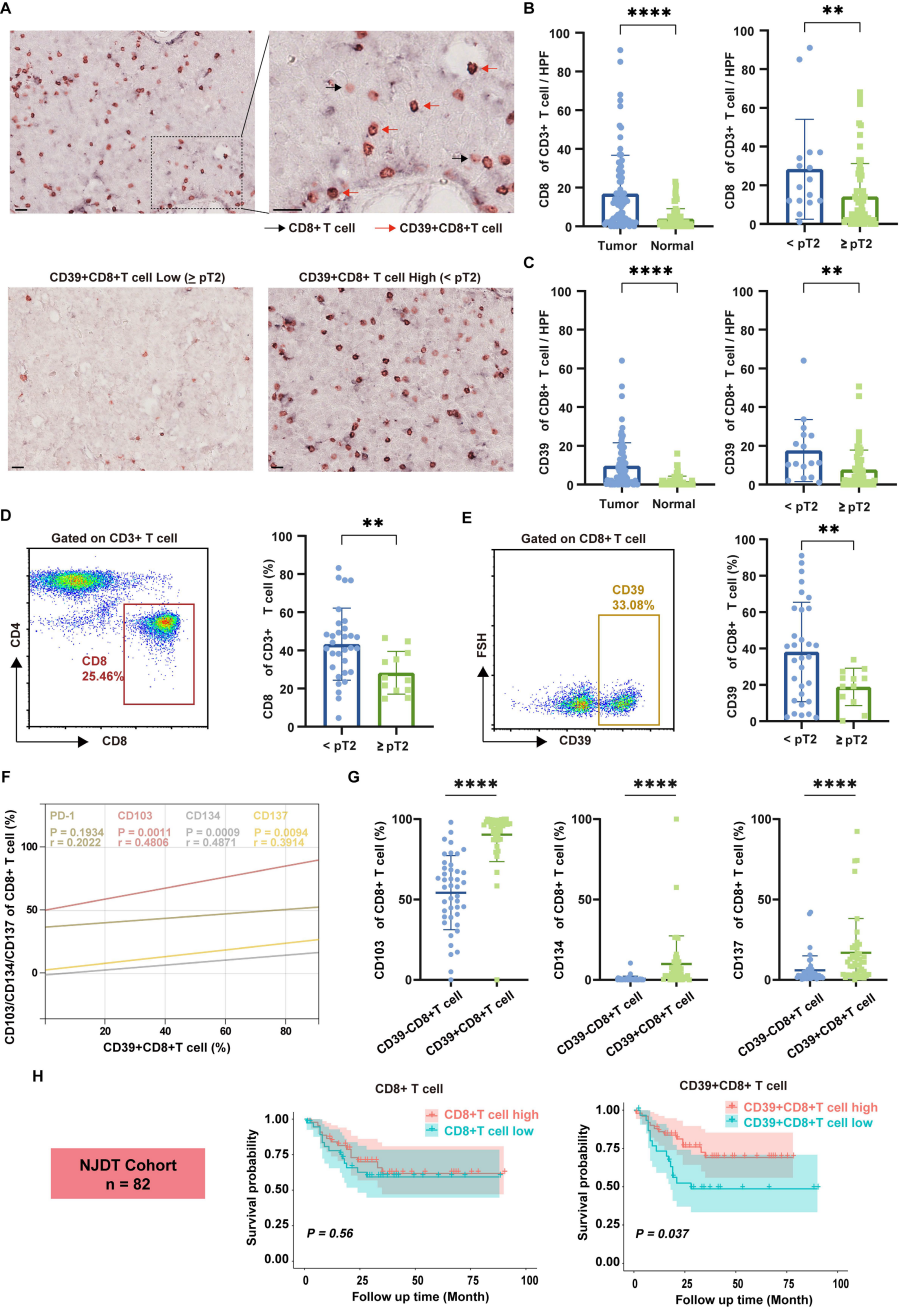
** Tumor-infiltrating CD39⁺CD8⁺ T cells are enriched in BLCA, display an effector-like phenotype, and associate with tumor stage and patient survival.** (A) Representative double-stained immunohistochemistry (IHC) image for CD39 (brown) and CD8 (pink) in tumor tissue slice. Black arrowheads indicate CD8^+^ T cell, red arrowheads indicate CD39^+^ CD8^+^ T cell. Scale bar, 20 μm. (B) Quantification of CD8⁺ T cell infiltration in tumor and normal bladder tissues. (C) Quantification of CD39⁺CD8⁺ T cell infiltration in tumor and normal bladder tissues. (D) Representative flow cytometry gating of CD8 expression of CD3^+^ T cells and quantification of CD8⁺ T cell frequencies across tumor stages. (E) Representative flow cytometry gating of CD39 expression of CD8^+^ T cells and quantification of CD39^+^CD8⁺ T cell frequencies across tumor stages. (F) Correlation between CD39 expression and PD-1, CD103, CD134, and CD137 expression on tumor-infiltrating CD8⁺ T cell. (G) Flow cytometric comparison of CD103, CD134, and CD137 frequencies between CD8⁺CD39⁻ and CD8⁺CD39⁺ T cells. (H) Kaplan-Meier analysis of overall survival stratified by intratumoral CD8^+^ T cell and CD39⁺CD8⁺ T cell infiltration in the NJDT cohort (n = 82). ** *P* < 0.01, **** *P* < 0.0001.

To identify the presence of CD39^+^CD8^+^ T cells in BLCA, we analyzed formalin-fixed and paraffin-embedded (FFPE) tissues through IHC staining and fresh tumor samples by flow cytometry, using a cohort from Nanjing Drum Tower Hospital (NJDT cohort). IHC staining revealed a significant increase in CD8⁺ T cell infiltration in tumor tissues compared to normal tissues, with a notable rise in lower-stage tumors (all* P* < 0.01, Figure 2A, 2B). Similarly, CD39⁺CD8⁺ T cell infiltration followed the same pattern, showing increased presence in tumor tissues, particularly in lower-stage tumors (all *P* < 0.01, Figure 2C).

Flow cytometry analysis of fresh bladder tumor specimens further corroborated these observations, demonstrating similar trends across different tumor stages (all *P* < 0.01, Figures 2D, 2E). Importantly, correlation analysis between CD39 and various immune-related markers shown that CD39 levels within CD8⁺ TILs were not significantly correlated with PD-1 (r = 0.2022, *P* = 0.1934). In contrast, markers of tissue residency and effector functions, including CD103 (r = 0.4806, *P* = 0.0011), CD134 (r = 0.4871, *P* = 0.0009), and CD137 (r = 0.3914, *P* = 0.0094), demonstrated a positive correlation with CD39 expression in CD8⁺ TILs (Figure 2F). The expression of CD103, CD134, and CD137 was higher in CD39⁺CD8⁺ T cells (all *P* < 0.0001, Figure 2G), indicating that CD39⁺CD8⁺ TILs may be enriched for a tissue-resident, activation-associated phenotype.

Further analysis of a cohort of 82 bladder cancer patients revealed that CD39⁺CD8⁺ T cell infiltration was significantly associated with OS, while total CD8⁺ T cell counts did not show a significant correlation with prognosis (Figure 2H). These findings preliminarily support the hypothesis that CD39⁺CD8⁺ TILs in BLCA patients possess an effector/activation-linked phenotype, and that their abundance tracks with a more favorable clinical outcome. Together, these data motivate a functional and clonotype-resolved evaluation of CD39⁺CD8⁺ TILs as a candidate tumor-reactive compartment.

### Tumor-infiltrating CD39^+^CD8^+^ T cells display an effector-like, tumor-reactive program in the murine MB49 model at single-cell resolution

**Figure 3:**
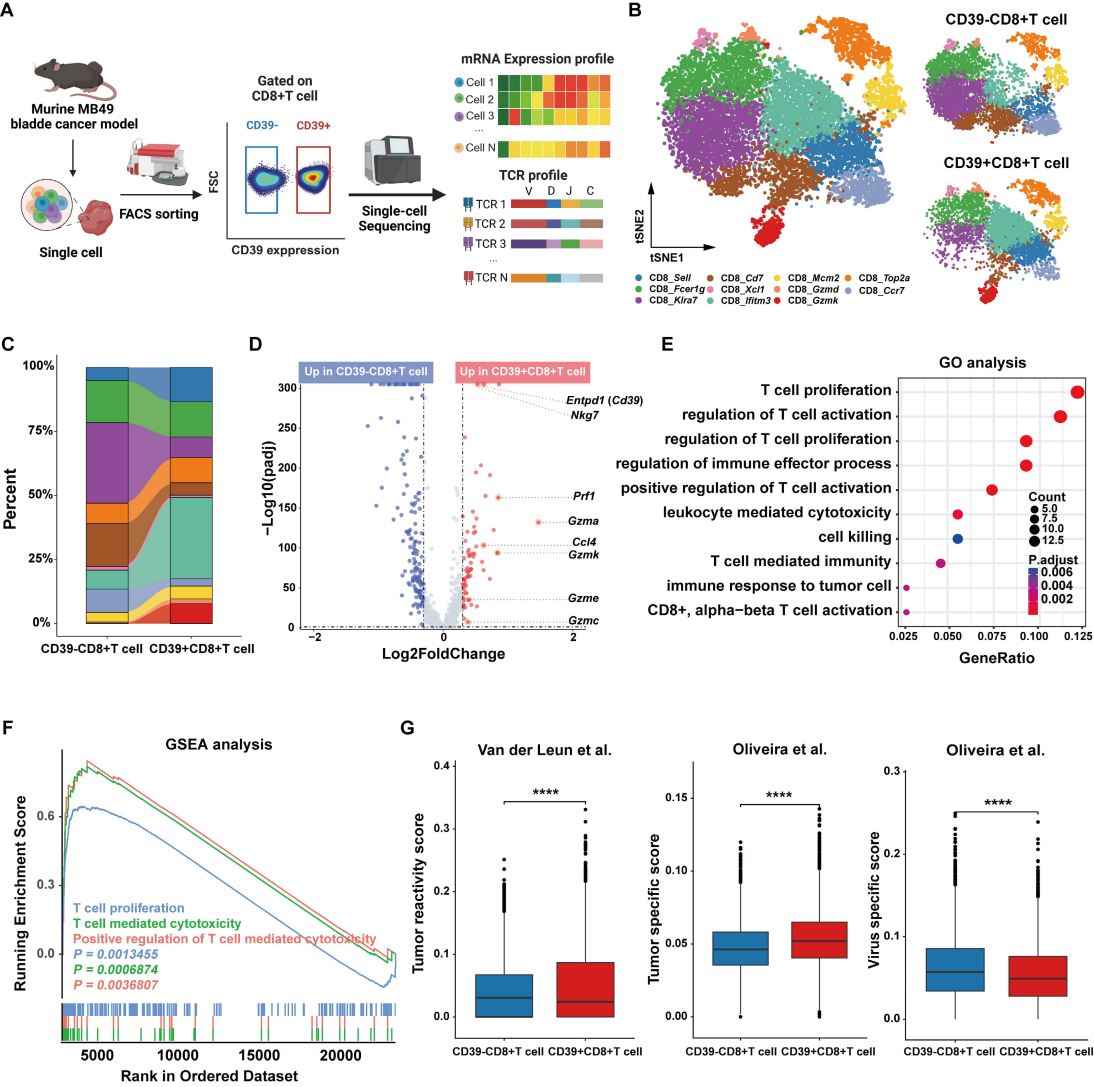
** Single-cell transcriptomic profiling identifies an effector-like program in CD39⁺CD8⁺ TILs from the murine MB49 model.** (A) Experimental workflow for FACS isolation of CD39⁺CD8⁺ TILs and CD39⁻CD8⁺ TILs from MB49 tumors followed by paired scRNA-seq and scTCR-seq. (B) t-SNE visualization of scRNA-seq data showing the distribution of CD39⁺CD8⁺ TILs and CD39⁻CD8⁺ TILs. (C) Comparison of the cell ratio of major cell types in CD39⁺CD8⁺ TILs and CD39⁻CD8⁺ TILs. (D) Volcano plot shows the differentially expressed genes between CD39^+^CD8^+^ TILs and CD39^-^CD8^+^ TILs. (E) GO enrichment analysis of genes upregulated in CD39⁺CD8⁺ TILs. (F) GSEA enrichment plots of T cell function-related genes upregulated in CD39⁺CD8⁺ TILs. (G) Signature scores for tumor reactivity, tumor specificity and virus-specific gene signatures. **** *P* < 0.0001.

To characterize the function of CD39 in CD8⁺ TILs, and given the absence of a reference genetic signature to define tumor-infiltrating CD39^+^CD8^+^ T cells in mice and the potential discrepancies between the transcript and protein expression levels of CD39, we therefore sorted CD39⁺CD8⁺ TILs and CD39⁻CD8⁺ TILs from MB49 tumor for scRNA-seq and scTCR-seq analysis (Figure 3A). After stringent quality control and filtering steps, transcriptomes from 5,665 CD39⁺CD8⁺ T cells and 6,735 CD39⁻CD8⁺ T cells were analyzed. Based on well-established CD8⁺ T cell markers, we classified the cells into eleven major subsets, including CD8_*Sell*, CD8_*Fcer1g*, CD8_*Kira7*, CD8_*Cd7*, CD8_*Xcl1*, CD8_*Iftm3*, CD8_*Mcm2*, CD8_*Gzmd*, CD8_*Gzmk*, CD8_*Top2a*, and CD8_*Ccr7* (Figure 3B).

Compared to CD39⁻CD8⁺ TILs, the CD8_*Kira7*, CD8_*Cd7*, and CD8_*Ccr7* subsets were significantly reduced, while CD8_*Top2a*, CD8_*Fcer1g*, CD8_*Mcm2*, and CD8_*Gzmk* were notably increased in CD39⁺CD8⁺ TILs (Figure 3C). This suggests that CD39⁺CD8⁺ TILs are enriched for proliferative and cytotoxic/effector-like states and depleted for subsets consistent with less-activated phenotypes. Volcano plot analysis revealed that effector-associated genes, including *Nkg7*, *Prf1*, *Gzma*, *Ccl4*, *Gzmk*, *Gzme* and *Gzmc*, were significantly enriched in the CD39⁺CD8⁺ T cell cluster (Figure 3D). GO and GSEA enrichment further confirmed the anti-tumor immunity potential of the CD39⁺CD8⁺ T cell population, with significant enrichment in pathways related to T cell-mediated cytotoxicity, T cell activation, and T cell proliferation (Figure 3E, 3F). Furthermore, CD39⁺CD8⁺ TILs exhibited higher transcriptomic signature scores for tumor reactivity and tumor specificity, and lower scores for virus-specific responses (all *P* < 0.0001, Figure 3G). These findings suggest that CD39⁺CD8⁺ TILs are enriched for effector phenotypic markers and are strongly associated with a tumor-reactive transcriptional program in the murine bladder tumor model.

### Clonotype-resolved analysis reveals tumor-infiltrating CD39⁺CD8⁺ T cell mediates anti-tumor immunity

**Figure 4:**
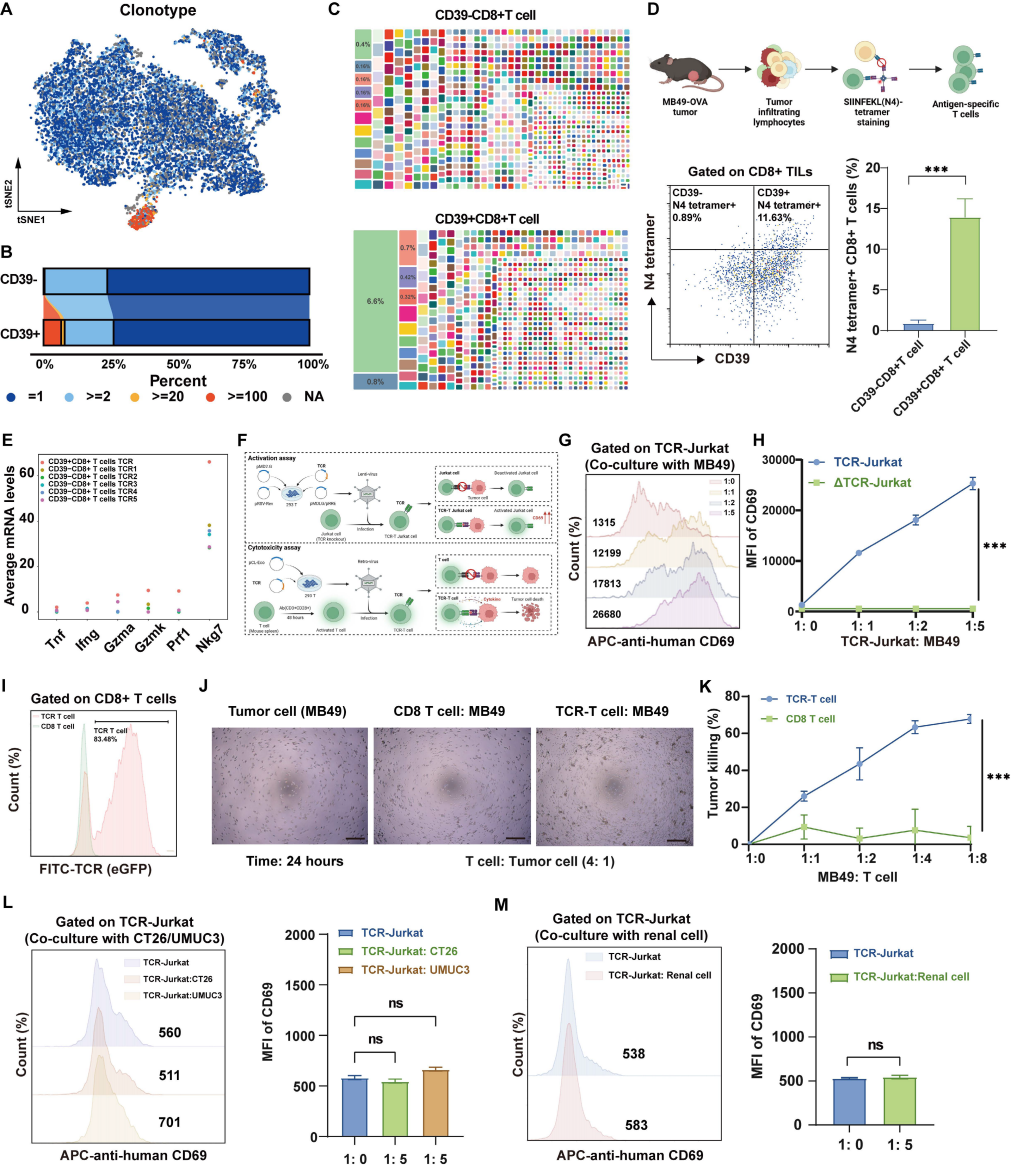
** Clonotype-resolved single-cell TCR analysis identifies tumor-reactive TCR sequence from CD39⁺CD8⁺ TILs.** (A) Distribution of TCR clonotypes from MB49 tumor. (B) Stacked chart shows TCR clone-size distribution of CD39^+/-^CD8^+^ T cells population. (C) Tree maps of TCR clonotypes in CD39^+/-^CD8^+^ TIL population. (D) Co-expression of CD39 and N4 tetramer among CD8^+^TILs in MB49-OVA tumor. (E) mRNA expression levels of *Tnf*, *Ifng*, *Gzma*, *Gzmk*, *Prf1* and *Nkg7* in the dominant TCR clonotype of CD39^+^CD8^+^ TILs and the top 5 TCR clonotypes from CD39^-^CD8^+^ TILs. (F) Scheme summarizes the activation and cytotoxicity assay of tumor reactive TCR. (G-H) Expression of CD69 on TCR-Jurkat cells after co-culture with MB49 tumor cells. (I) Viral infection efficiency of TCR-T cells. (J) Representative microscopy image after 24 hours co-culture of TCR-T cells with MB49 tumor cells. (K) Tumor cell killing by TCR-T cells in co-culture with MB49 cells. (L) TCR-Jurkat co-cultured with tumor cells of different MHC background, including CT26 (H-2K^d^) or UMUC3 (HLA-A*02). (M) TCR-Jurkat co-cultured with normal renal cells. ns, *P* > 0.05, *** *P* < 0.001.

Tumor-specific recognition by TCRs is the basis of the anti-tumor function of tumor-reactive T cells, and we therefore profiled the TCR repertoire of CD39⁺ CD8⁺ TILs and CD39⁻CD8⁺ TILs (Figure 4A). Clonal composition of T cells was classified by clone size (NA = no TCR detected, ≥2, ≥20, ≥100). CD39⁺CD8⁺ T cells displayed TCR clonal expansion, particularly in clone size ≥ 20 (Figure 4B). The dominant TCR clonotype in CD39⁺CD8⁺ T cells accounted for 6.6% of the total clones, compared to only 0.4% in CD39⁻CD8⁺ T cells (Figure 4C). Next, we examined the expression of CD39 on OVA-derived antigen (SIINFEKL, N4) specific T cells within the TIME of an antigen-known system (the syngeneic MB49-OVA tumor model) (Figure S3). Nearly all N4 tetramer⁺ T cells expressed CD39, with less than 1% being CD39 negative, supporting CD39 as a valuable surface biomarker for identifying tumor cell-derived antigen-specific CD8⁺ T cells. Since SIINFEKL is the dominant antigen in the syngeneic MB49-OVA tumor model, thus the TCR clone frequency targeting this antigen reaches 15% in CD39⁺CD8⁺ TILs, and the remaining CD8⁺CD39⁺ T cells may possess the potential to recognize other MB49 tumor-derived antigens (Figure 4D).

Moreover, comparison of the top five TCR clones in CD39⁻CD8⁺ TILs with those in CD39⁺CD8⁺ TILs revealed that the dominant CD39⁺ clones expressed markedly higher levels of effector-related genes, such as *Ifng* and *Nkg7*, indicating a cytotoxic transcriptional program (Figure 4E). To further investigate the functional relevance of these findings, we successfully obtained the full TCR sequence and generated TCR-Jurkat cells and TCR-T cells based on the most abundant TCR in CD39⁺CD8⁺ TILs (Figure 4F, Figure S4). TCR-Jurkat cells recognizing MB49 tumor cells exhibited tumor antigen-dependent activation, with a significant increase in the activation marker CD69 upon MB49 tumor cell stimulation, compared to TCR-knockout (ΔTCR) Jurkat cells (Figure 4G, 4H). TCR-T cells also demonstrated significant tumor-specific killing ability after 24 hours of co-culture with MB49 tumor cells, even at a lower ratio (Figure 4I-4K). More importantly, TCR-Jurkat cells were not activated following co-culture with tumor cells with mismatched MHC haplotypes, including mouse colorectal cancer CT26 cells (H-2K^d^) and human bladder cancer UMUC3 cells (HLA-A*02), as evidenced by no significant increase in CD69 (*P* > 0.05, Figure 4L). Furthermore, CD69 remained uninduced on TCR-Jurkat cells following co-culture with normal renal cells (*P* > 0.05, Figure 4M). Together, these results support MHC-restricted, tumor-selective recognition by CD39⁺CD8⁺ TIL-derived TCR and underscore the translational potential of CD39⁺CD8⁺ TILs as an effective and safe therapeutic cell population.

### Adoptive transfer of tumor-infiltrating CD39⁺CD8⁺ T cells is feasible and safe

**Figure 5:**
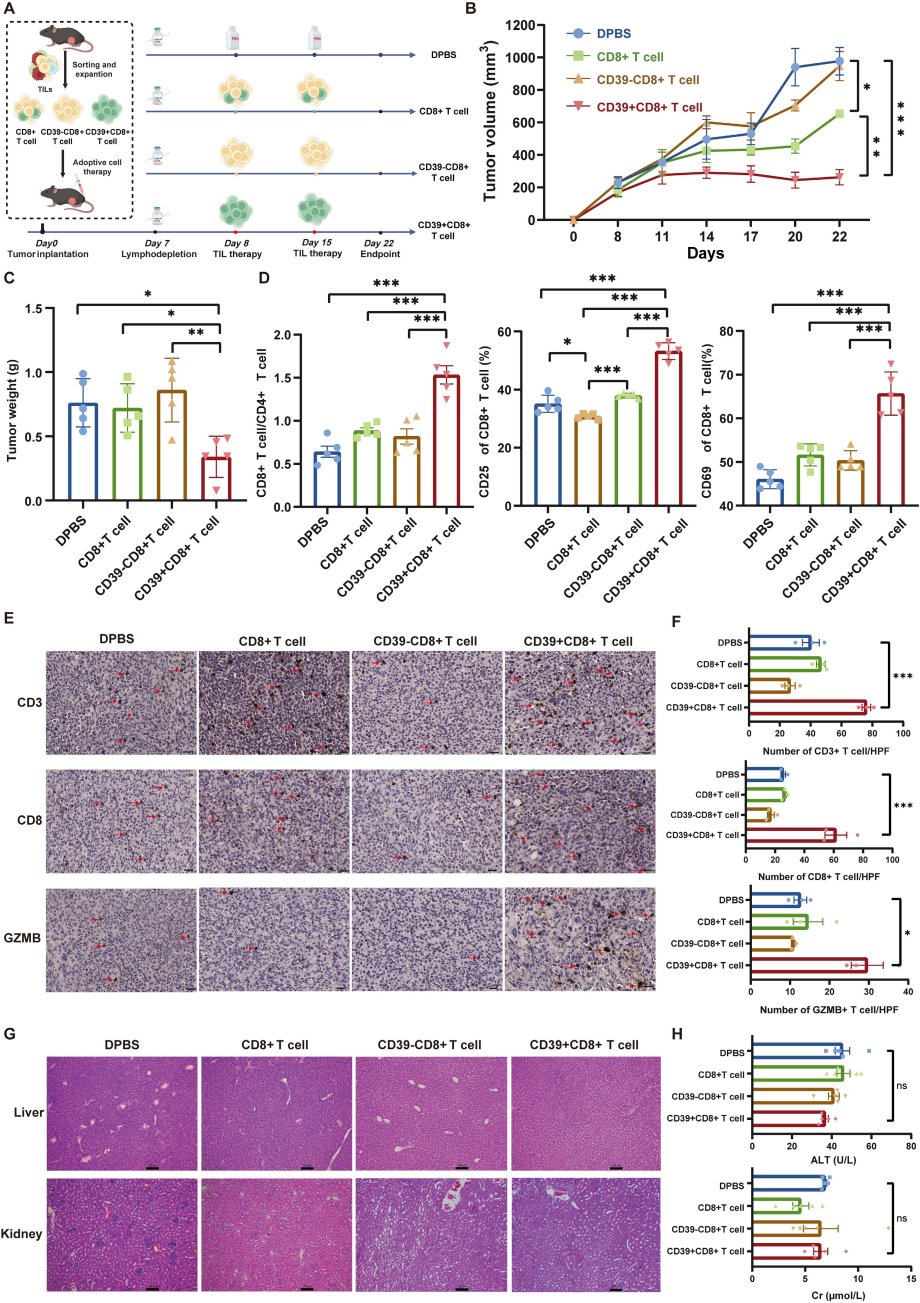
** Adoptive transfer of CD39⁺CD8⁺ TILs suppresses MB49 tumor growth with a favorable safety profile.** (A) Experimental design and treatment schedule for systemic adoptive cellular therapy using CD8^+^ TILs, CD39⁻CD8⁺ TILs, or CD39⁺CD8⁺ TILs (n = 5). (B) Tumor growth curves of MB49 tumors under the indicated treatments. (C) Weights of excised tumors at endpoint. (D) Flow cytometry analysis showing the percentage of CD8^+^/CD4^+^ T cells, CD25^+^CD8^+^ T cells, and CD69^+^CD8^+^ T cells within different treatments. (E) Representative IHC images for infiltration of CD3, CD8, and GZMB positive cells in four groups. (F) Quantification of CD3⁺, CD8⁺, and GZMB⁺ cells per field of view under identical imaging conditions. (G) Representative H&E staining for liver, lung and kidney. Bar = 100 μm, (H) Blood tests for liver and renal function. ns, *P* > 0.05, * *P* < 0.05; ** *P* < 0.01; *** *P* < 0.001.

To further validate the therapeutic potential of CD39⁺CD8⁺ TILs, we sorted and expanded CD8⁺ TILs, CD39⁻CD8⁺ TILs, and CD39⁺CD8⁺ TILs from MB49 bladder tumors and transferred these populations into a syngeneic mouse model to treat MB49 bladder tumors (Figure 5A). Compared to the DPBS group, as well as the CD8⁺ T cell and CD39⁻CD8⁺ T cell groups, CD39⁺CD8⁺ T cell treatment resulted in the smallest tumor sizes and weights (*P* < 0.05, Figures 5B, 5C). Notably, tumors in mice treated with CD39⁺CD8⁺ T cells displayed minimal progression over time, whereas CD39⁻CD8⁺ T cell treatment failed to suppress tumor growth (Figure 5B). Flow cytometry analysis revealed a significant increase in the CD8⁺ T cell/CD4⁺ T cell ratio following treatment with CD39⁺CD8⁺ TILs, with a higher percentage of CD8⁺ TILs being activated (*P* < 0.001, Figure 5D). IHC staining shown that reinfusion of CD39⁺CD8⁺ TILs not only promoted infiltration of CD3⁺ T cells and CD8⁺ T cells into the tumors but also increased the expression of GZMB, a serine protease released by cytotoxic T cells (*P* < 0.05, Figures 5E, 5F).

After confirming the therapeutic efficacy of CD39⁺CD8⁺ TILs, and observing no treatment-related mortality or overt signs of toxicity, we assessed the safety of this therapy. H&E staining and histopathological analysis revealed no abnormalities in the liver and renal tissues of mice treated with CD39⁺CD8⁺ TILs (Figure 5G). Additionally, blood serum tests indicated that CD39⁺CD8⁺ TIL therapy had a negligible impact on liver and renal function (*P* > 0.05, Figure 5H). Levels of alanine aminotransferase (ALT) and creatinine (Cr) in the CD39⁺CD8⁺ T cell group remained within reference intervals, further supporting the safety of this treatment. These findings suggest that the reinfusion of tumor-infiltrating CD39⁺CD8⁺ T cells is not only therapeutically effective but also well tolerated in this syngeneic model, supporting CD39⁺CD8⁺ TILs as a feasible cell source for ACT in solid tumors.

### Tumor-infiltrating CD39^+^CD8^+^ T cells exhibit the potential for anti-tumor immunity in BLCA patients

**Figure 6:**
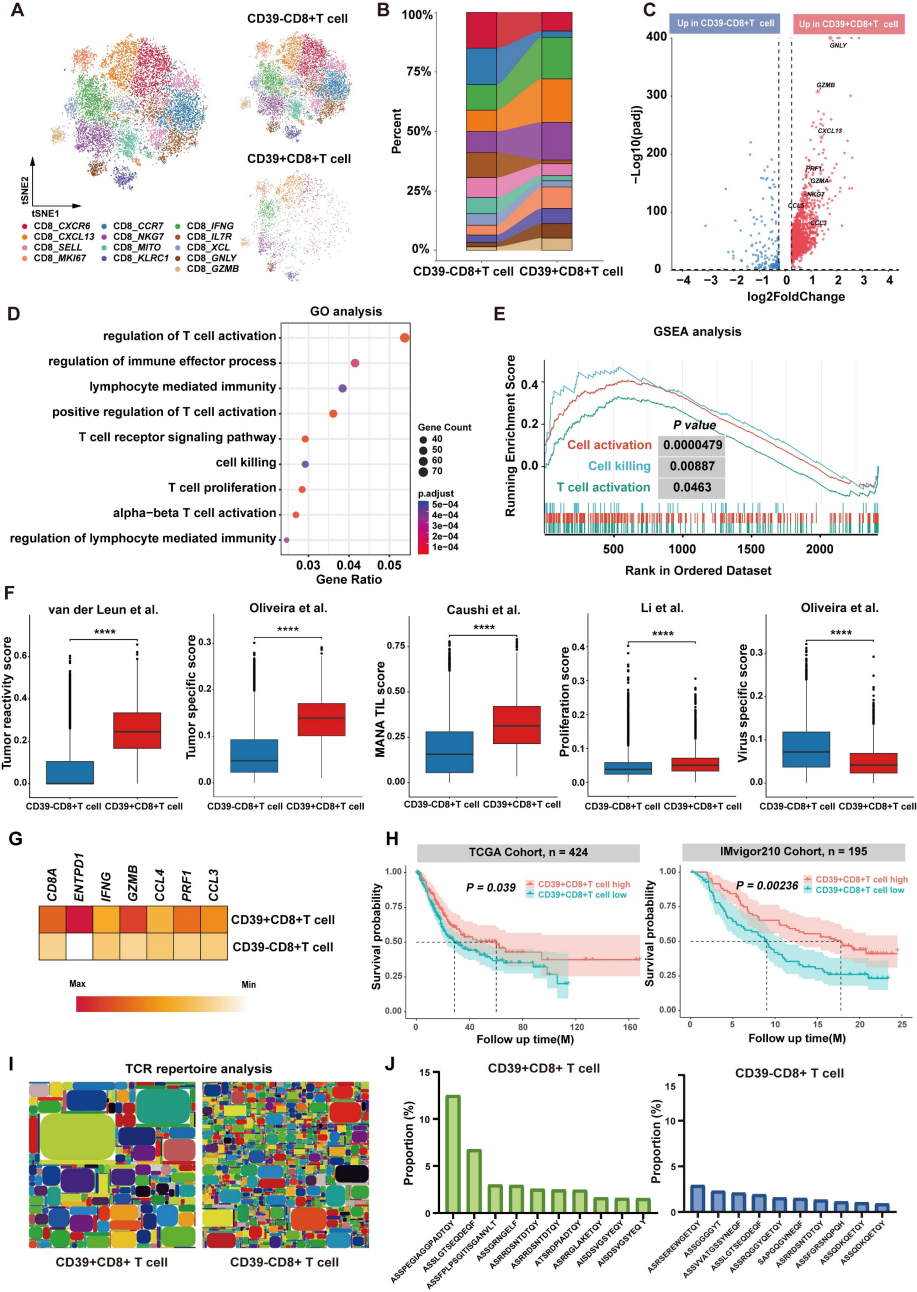
** Single-cell transcriptional profile and bulk TCR repertoire of human bladder cancer.** (A) t-SNE visualization of integrated scRNA-seq data showing CD39⁺CD8⁺ TILs and CD39⁻CD8⁺ TILs. (B) Comparison of the cell ratio of major cell types in CD39⁺CD8⁺ TILs and CD39⁻CD8⁺ TILs. (C) Volcano plot shown the differentially expressed genes between CD39⁺CD8⁺ TILs and CD39⁻CD8⁺ TILs. (D) GO enrichment analysis of genes upregulated in CD39⁺CD8⁺ TILs. (E) GSEA enrichment plots of T cell function-related genes upregulated in CD39⁺CD8⁺ TILs. (F) Signature scores for tumor reactivity, tumor specificity, mutation-associated neoantigen (MANA) TIL, proliferation and virus-specific gene signatures. (G) Expression of CD39^+^CD8^+^ TIL signature-related genes (G) Kaplan-Meier curves for overall survival stratified by CD39⁺CD8⁺ TIL signature enrichment in the TCGA-BLCA cohort (n = 424) and the IMvigor210 cohort (n = 195). (H) Tree maps of TCR clonotypes in CD39⁺CD8⁺ TILs and CD39⁻CD8⁺ TILs. (I) Relative frequencies of the top 10 most abundant TCR clonotypes. **** P < 0.0001.

To validate the clinical relevance of our findings, we performed pairwise comparisons between CD39⁺CD8⁺ TILs and CD39⁻CD8⁺ TILs using scRNA-seq. We integrated scRNA-seq data from the GSE222315, GSE146137, GSE130001, GSE149652, and GSE176249 datasets (20 patients) and our own cohort (3 patients) using a batch effect correction algorithm, which included data from 16,785 CD8⁺ T cells. Based on marker gene signatures, we annotated the cells into CD39⁺CD8⁺ T cells and CD39⁻CD8⁺ T cells for further analysis. t-SNE analysis was identified 13 major CD8^+^ T cell types, including CD8_*CXCR6*, CD8_*CCR7*, CD8_*IFNG*, CD8_*CXCL13*, CD8_*NKG7*, CD8_*IL7R*, CD8_*SELL*, CD8_*MITO*, CD8_*XCL*, CD8_*MKI67*, CD8_*KLRC1*, CD8_*GNLY*, and CD8_*GZMB*, (Figure 6A). Compared to CD39⁻CD8⁺ TILs, CD8_*IFNG*, CD8_*CXCL13*, CD8_*NKG7*, CD8_*MKI67*, CD8_*KLRC1*, CD8_*GNLY*, and CD8_*GZMB* were enriched in the CD39⁺CD8⁺ TIL population, which indicated heightened cytotoxic, proliferative, and effector programs. CD8_*CXCR6*, CD8_*CCR7*, CD8_*IL7R,* CD8_*MITO,* and CD8_*XCL* were reduced in CD39⁺CD8⁺ TILs, which were mainly associated with the memory and metabolic states of T cells (Figure 6B). Further differential gene expression analysis revealed that CD39⁺CD8⁺ TILs exhibited enrichment of effector T cell-related genes, including *GZMB*, *CXCL13*, *NKG7*, and *GNLY*, and were significantly associated with pathways such as positive regulation of T cell activation, T cell receptor signaling pathway, and T cell-mediated immunity (Figure 6C-6E). The transcriptomic signature scores for tumor reactivity, tumor specificity, mutation-associated neoantigens TIL and proliferation were higher in CD39⁺CD8⁺ TILs, whereas the virus-specific score was lower (all *P* < 0.001, Figure 6F).

We next examined the prognostic significance of CD39⁺CD8⁺ TIL in external cohorts according to the feature signature genes, including *CD8A*, *ENTPD1*, *IFNG*, *GZMB*, *CCL4*, *PRF1*, and *CCL3* (Figure 6G). The analysis included 619 patients from the TCGA-BLCA cohort (n = 424) and the IMvigor210 cohort (ICI-treated bladder cancer patients, n = 195). We found that higher CD39⁺CD8⁺ TIL signature enrichment was a significant predictor of OS across all cohorts. Specifically, higher signature enrichment of CD39⁺CD8⁺ TILs was associated with better OS in both the TCGA-BLCA cohort (*P* = 0.00092) and the IMvigor210 cohort (*P* = 0.034) (Figure 6H). Moreover, bulk TCR sequencing analysis revealed that CD39⁺CD8⁺ TILs had lower TCR clonal diversity and higher TCR clonal expansion compared to CD39⁻CD8⁺ TILs (Figure 6I). The most abundant TCR clone constituted approximately 13% of CD39⁺CD8⁺ TILs, compared to only 3% in CD39⁻CD8⁺ TILs, further supporting the tumor-reactivity of CD39⁺CD8⁺ TILs (Figure 6J). These results indicate that CD39⁺CD8⁺ TILs play a crucial role in anti-tumor immunity in BLCA patients and likely represent a tumor antigen-reactive T cell subset, consistent with our preclinical findings, and support CD39 as a practical marker to enrich tumor-reactive CD8⁺ TILs for adoptive cell therapy to improve clinical outcomes of solid tumors.

## Discussion

TIL therapy, first applied in melanoma in the 1980s, has re-emerged with modern manufacturing and immunotherapy strategies and now shows meaningful activity across solid tumors 40-43. BLCA is similarly immunogenic, with among the highest tumor mutational burdens, and has a long clinical history with immunotherapy, ranging from intravesical Bacillus Calmette-Guérin (BCG) in non-muscle-invasive bladder cancer (NMIBC) to ICIs in muscle-invasive bladder cancer (MIBC) 44, 45. Although these characteristics make bladder cancer as a potential candidate for adoptive cell therapy, the widespread issue of insufficient numbers and dysfunction of tumor-reactive T cells has hindered the application of TIL therapy in the treatment of bladder cancer. Typically, only a small fraction of TILs is tumor reactive, whereas bystander T cells expand more readily during *ex vivo* culture, thereby diluting therapeutic potency^15^, ^46^, ^47^. Strategies that prospectively enrich tumor-reactive T cells before expansion are therefore needed.

Here, using clonotype-resolved single-cell multi-omics, we systematically re-evaluated the biological and therapeutic value of CD39⁺CD8⁺ TILs and tested their feasibility both as an ACT source population and as a stratification biomarker. CD39⁺CD8⁺ TILs exhibited reinforced activation and cytotoxic transcriptional programs, together with pronounced TCR clonal expansion. Tumor reactivity and specificity were corroborated by TCR-Jurkat activation and TCR-T cell cytotoxicity assays. *In vivo*, adoptive transfer of prospectively isolated CD39⁺CD8⁺ TILs achieved significant tumor control with a favorable safety profile. Clinically, CD39⁺CD8⁺ TILs infiltration, rather than bulk CD8⁺ TILs density, was associated with earlier tumor stage, prolonged OS, and improved response to ICI across multiple BLCA cohorts. Together, these results position CD39 as a practical enrichment handle to increase the tumor-reactive content of ACT cell preparations in BLCA.

CD39 (ENTPD1) sits at the apex of the ATP-adenosine axis in the TIME: extracellular ATP released under stress or tumor cell death is hydrolyzed by CD39 to AMP and further converted by CD73 to adenosine, which dampens immune response via A2A/A2B signaling^39^. While this metabolic pathway underpins the canonical immunosuppressive role of CD39^48^, ^49^, CD39 expression correlates with clinical outcomes following T cell-targeted PD-1/CTLA-4 blockade but not tumor cell-targeted PD-L1 blockade, highlighting the cell-type-specific functional heterogeneity of CD39. Recent evidence suggests a dual role for CD39, implicating it not only in immunosuppression but also as a potential biomarker of tumor reactivity in esophageal squamous cell carcinoma and clear cell renal cell carcinoma^27^, ^28^, ^50^. Using TCR clonotype-resolved single-cell multi-omics analyses, we validated the tumor reactivity of CD39^+^CD8^+^ TILs in bladder cancer. Within CD8⁺ TILs, CD39 defines a subset coupled to tumor recognition, as evidenced by clonal expansion, effector-competent programs, and tumor-killing function. Importantly, we show that this population can be prospectively isolated and expanded *ex vivo* under supportive conditions while preserving tumor recognition capacity while mitigating potential immunosuppressive effects. Moreover, consistent with our clinical evidence, increases in CD39⁺CD8⁺ T cells have been linked to better outcomes or heightened immune responses in multiple settings, including neoadjuvant atezolizumab in cisplatin-ineligible MIBC^24^-^26^, ^51^-^53^. Together, these observations support CD39-guided enrichment as a practical route to increase the tumor-specific content of ACT cell preparations while reducing the expansion of irrelevant or suppressive T cells.

However, several limitations of this study need to be addressed in future research. First, mechanistic and *in vivo* validation relied primarily on the MB49 syngeneic model and human BLCA datasets. Larger and more diverse clinical cohorts are needed to validate our findings and confirm the generalizability of these results. Second, while CD39 is a powerful selector of tumor-reactive CD8⁺ T cells, the precise mechanisms underlying the role of CD39 in modulating T cell function in the TIME remain unclear. Future studies should explore the long-term persistence and differentiation of CD39⁺CD8⁺ TILs post-reinfusion, as well as investigate potential synergistic effects with other immunotherapies, such as ICIs or targeted therapies. Third, broader validation across additional tumor models and a larger panel of CD39⁺CD8⁺ TIL-derived TCRs will be important to define the sensitivity and specificity of CD39 as an enrichment marker for tumor reactivity.

## Conclusion

Our study provided evidence that CD39⁺CD8⁺ TILs represent a critical population in the tumor immune landscape and have significant potential as a practical biomarker to enrich tumor-reactive T cells for adoptive cell therapy. These results not only provide valuable insights into the immunological role of CD39 but also highlight the promise of CD39⁺CD8⁺ TILs in enhancing the efficacy of cancer immunotherapies.

## Supplementary Material

Supplementary figures and tables 1-3.

Supplementary table 4.

## Data Availability

The raw FASTQ data for mouse CD39⁺CD8⁺ TILs and CD39⁻CD8⁺ TILs have been deposited into the National Genomics Data Center (PRJCA056527). Other datasets used during the current study are available from the corresponding author on reasonable request.

## Abbreviations

ICIs: immune checkpoint inhibitors
TILs: tumor-infiltrating lymphocytes
TCR-pMHC: T cell receptor-peptide-major histocompatibility complex
ACT: adoptive cell therapy
TIME: tumor immune microenvironment
eATP: extracellular ATP
BLCA: bladder cancer
OS: overall survival
FBS: fetal bovine serum
H&E: hematoxylin and eosin
IHC: immunohistochemistry
ΔTCR-Jurkat: knockout TCR Jurkat
IL-2: interleukin-2
scRNA-seq: single-cell RNA sequencing
scTCR-seq: single-cell TCR sequencing
cDNA: complementary DNA
HPF: high-power field
HVGs: highly variable genes
PCA: principal component analysis
t-SNE: t-distributed stochastic neighbor embedding
DEGs: differentially expressed genes
GO: Gene Ontology
GSEA: Gene Set Enrichment Analysis
CCA: Canonical Correlation Analysis
FFPE: formalin-fixed and paraffin-embedded
ALT: alanine aminotransferase
Cr: creatinine
BCG: Bacillus Calmette-Guérin
NMIBC: non-muscle-invasive bladder cancer
MIBC: muscle-invasive bladder cancer
