# Supplementary material for: Clonotype-Resolved Single-Cell Multi-Omics Unlocks the Profile of Tumor-Infiltrating CD39⁺CD8⁺ T Cells and Enables Adoptive Cell Therapy for Solid Tumor
Source: Int J Biol Sci. 2026 Feb 18;22(6):2869–84. doi: 10.7150/ijbs.130389 (PMC13050436; doi:10.7150/ijbs.130389)
Supplement: Supplementary file 1 — Supplementary figures and tables 1-3. [file ijbsv22p2869s1.pdf]

Supplementary Information

Clonotype-Resolved Single-Cell Multi-Omics Unlocks the Profile of Tumor-Infiltrating $CD39^{+}CD8^{+}$ T Cells and Enables Adoptive Cell Therapy for Solid Tumor

Authors: Zihan Zhao[1†], Xiangyu Wu[1†], Qingyang Jin[1†], Xin Yang[1], Wenjie Zhu[1], Ning Jiang[1], Tianyao Liu[3,1], Tianhang Li[1], Feng Fang[2]*, Hongqian Guo[1]*, Rong Yang[1]*

Affiliations: [1]Department of Urology, Nanjing Drum Tower Hospital, Affiliated Hospital of Medical School, Nanjing University, Nanjing, 210008, China

[2]Department of Pharmacology, School of Basic Medical Sciences, Nanjing Medical University, Nanjing, 211166, China

[3]Department of Genitourinary Oncology, Tianjin Medical University Cancer Institute and Hospital, National Clinical Research Center for Cancer, Key Laboratory of Cancer Prevention and Therapy, Tianjin, Tianjin's Clinical Research Center for Cancer, Tianjin, 300060, China

Correspondences: Rong Yang, E-mail: yangr@nju.edu.cn

Hongqian Guo, E-mail: dr.ghq@nju.edu.cn

Feng Fang, E-mail: fengfang@njmu.edu.cn

Keywords: CD39 (ENTPD1), tumor-infiltrating lymphocyte, TIL therapy, adoptive cell therapy, single-cell sequencing analysis, bladder cancer

†These authors have contributed equally to this work and share first authorship.

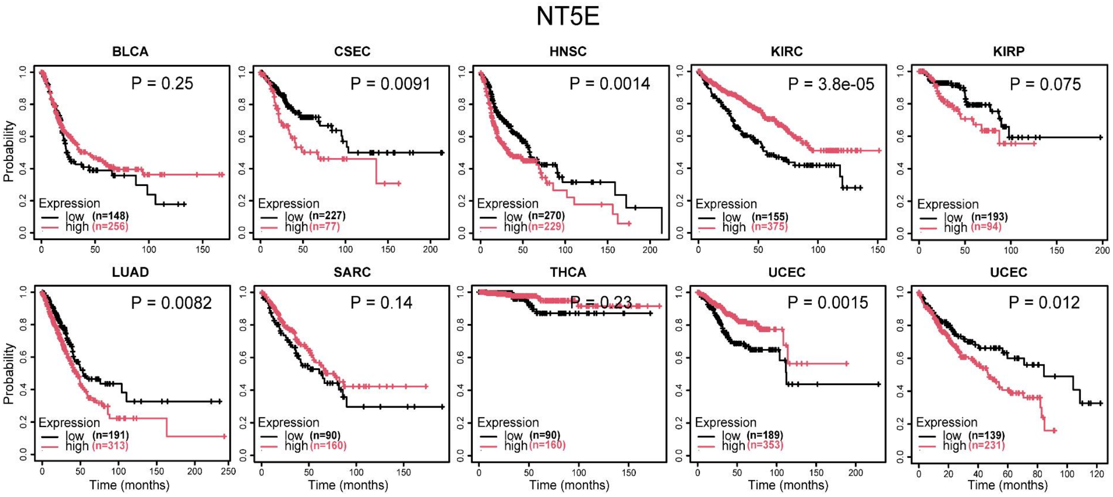

Figure S1. Kaplan-Meier curves for overall survival stratified by NT5E (CD73) expression across various solid tumors in the TCGA database.

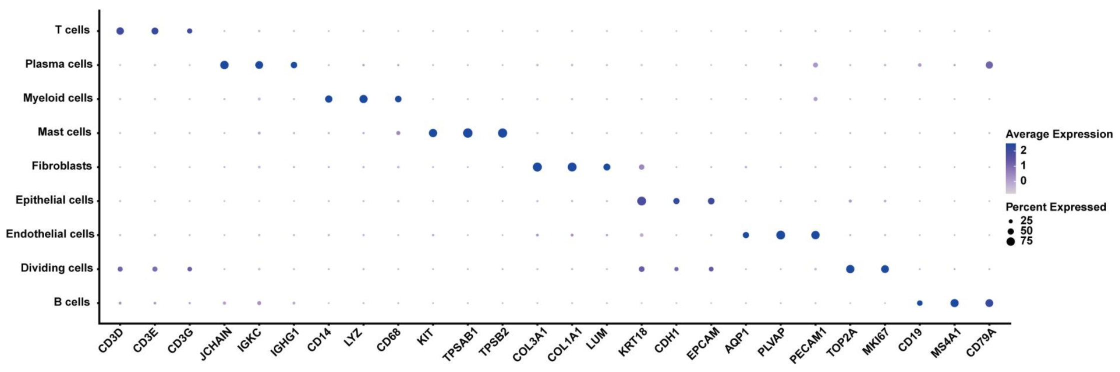

Figure S2. Top marker genes of each cell type in human bladder tissues.

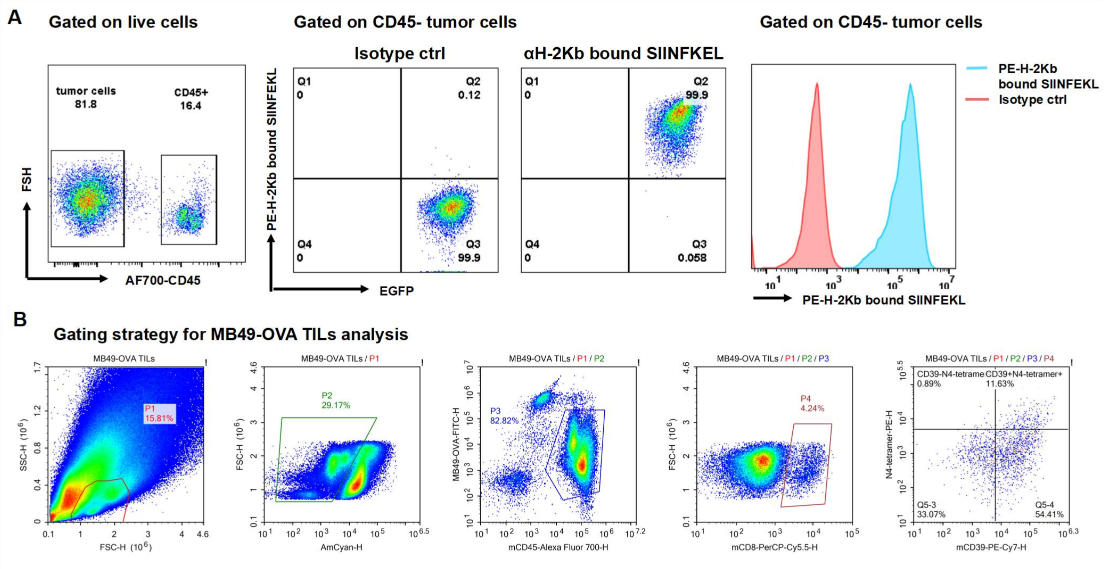

Figure S3. Establishment of MB49-OVA tumor cells and analysis of MB49-OVA TILs. (A) Expression levels of OVA in the MB49-OVA tumor cell. (B) Gating strategy for MB49-OVA CD8^{+}TILs analysis.

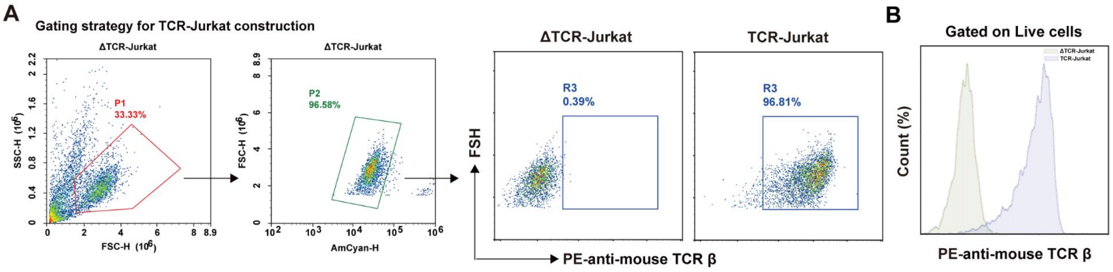

Figure S4. Construction of TCR-Jurkat cell. (A) Gating strategy for TCR-Jurkat construction. (B) Expression of mouse TCR β chain of TCR-Jurkat cell.

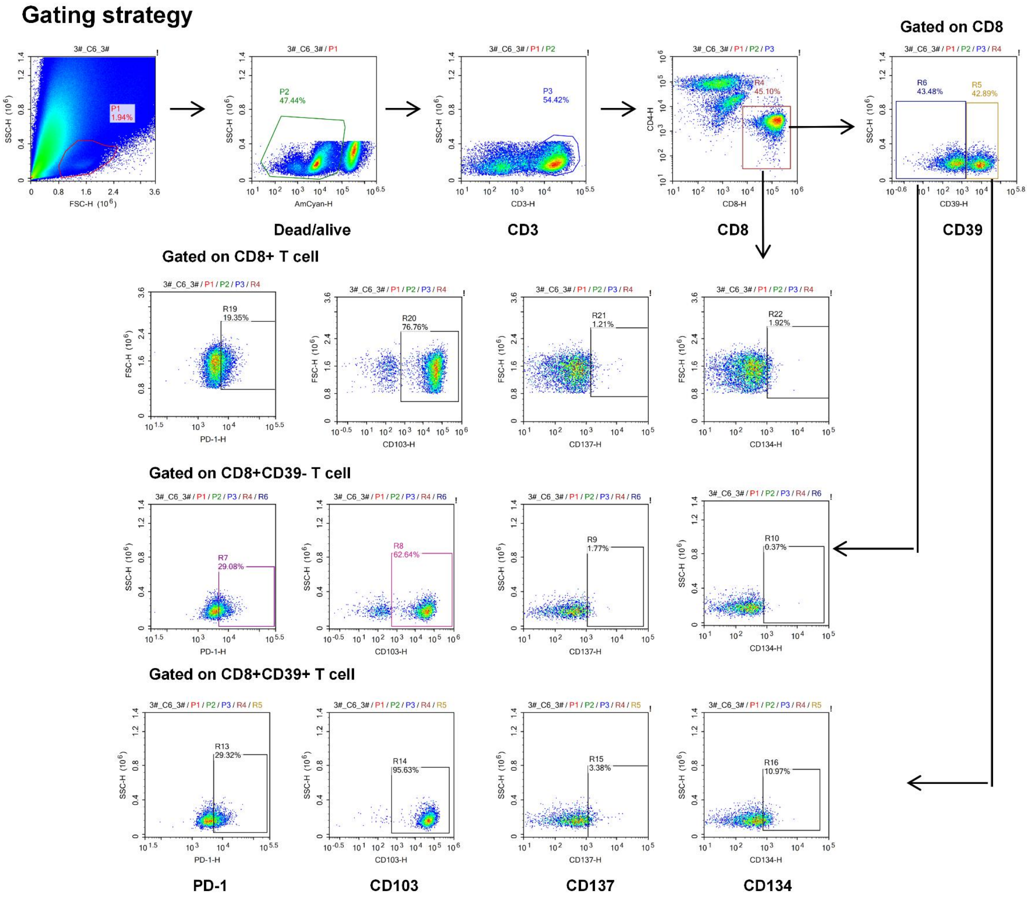

Figure S5. Representative flow cytometric gating strategy for $CD39^{+}CD8^{+}$ TILs and $CD39^{-}CD8^{+}$ TILs.

Table S1. Clinicopathologic parameters for the NJDT cohort of 82 patients with BLCA.

Parameter		Count	CD8+ TIL high	$CD39^{+}CD8^{+}$TIL high
**Age**	Young (< 67years)	17 (21%)	6 (35%)	13 (76%)
	Old (≥ 67years)	65 (79%)	33 (51%)	38 (58%)
**Gender**	Male	70 (85%)	34 (49%)	40 (57%)
	Female	12 (15%)	8 (67%)	11 (92%)
**Grade**	High	73 (89%)	39 (53%)	44 (52%)
	Low	5 (6%)	1 (20%)	5 (100%)
	Others	4 (5%)	3 (75%)	2 (50%)
**pT stage**	< T2	15 (18%)	13 (87%)	12 (80%)
	≥T2	66 (81%)	36(55%)	28 (42%)
	Tx	1 (1%)	1 (100%)	1 (100%)

Table S2. Clinicopathologic parameters of 43 BLCA patients for FACS analysis.

Parameter		Count
**Age**	Young (< 67years)	23 (53%)
	Old (≥ 67years)	20 (47%)
**Gender**	Male	37 (86%)
	Female	6 (14%)
**Grade**	High	31 (72%)
	Low	12 (28%)
**pT stage**	< T2	31 (72%)
	≥T2	12 (28%)

Table S3. Antibodies information for flow cytometry.

Antibody	Conjugate/Tag	Cat#
anti-human CD3	AF700	300324
anti-human CD4	PB	317429
anti-human CD8	PE	300908
anti-human PD-1	AF-647	329904
anti-human CD39	BV 605	328236
anti-human CD103	APC	350216
anti-human CD134 (OX40)	BV711	350030
anti-human CD137 (4-1BB)	APC/CY7	309830
